# In Vitro Comparative Evaluation of Frictional Resistance of Connecticut New Arch Wires, Stainless Steel and Titanium Molybdenum Alloy Archwires Against Different Brackets

**DOI:** 10.7759/cureus.6131

**Published:** 2019-11-12

**Authors:** Shraddha Suryavanshi, Uma Lingareddy, Nausheer Ahmed, Kiran Kumar Neelakantappa, Nishat Sidiqha, Manisha Minz

**Affiliations:** 1 Orthodontics and Dentofacial Orthopedics, Government Dental College and Research Institute, Bengaluru, IND; 2 Orthodontics and Dentofacial Orthopedics, Academic Unit of Dentistry, Bengaluru, IND; 3 Endodontics, Government Dental College and Research Institute, Bengaluru, IND; 4 Prosthodontics, Government Dental College and Research Institute, Bengaluru, IND

**Keywords:** friction, cna wire, surface roughness

## Abstract

Background and objectives

Friction between the bracket and archwire during sliding mechanics is of great concern in orthodontics, as it reduces the effectiveness of the orthodontic appliance and slows down tooth movement. The aim of this study was to evaluate frictional resistance of stainless steel (SS), titanium molybdenum alloy (TMA), and Connecticut new arch (CNA) wires against SS and ceramic brackets. The surface textures of the brackets and wires were also evaluated by scanning electron microscopy (SEM) before and after testing.

Method

A total of 180 premolar brackets of SS (ORMCO Corp., Orange, CA) and 180 ceramic (3M Unitek, Maplewood, MN) with a 0.022-inch slot and 180 SS, TMA, and CNA wires of 0.017 x 0.025 inches and 0.019 x 0.025 inches were tested. The SS brackets and ceramic brackets were bonded onto the SS bar with cyanoacrylate adhesive with the help of a jig. The wire assembly was vertically mounted and clamped to the jaws of the universal testing machine with 10 N load cell, and friction was measured along with other readings. The surface roughness of brackets and wires were examined using SEM in 200 X magnification before and after testing.

Results

TMA wire showed the greatest frictional force compared to SS and CNA wire. The frictional force was greater in the 0.019 x 0.025-inch wire compared to the 0.017 x 0.025-inch wires. The highest frictional force was noted in the SS bracket and 0.019 x 0.02-inch TMA wire combination. A statistically significant difference was not seen between the SS bracket and 0.019 x 0.025-inch SS wire and the 0.019 x 0.025-inch CNA wire combinations.

SEM showed that the TMA archwire had the roughest surface area compared to SS and CNA wires, and the ceramic bracket had more surface roughness than the SS bracket.

Conclusion

CNA wire demonstrated frictional resistance similar to the SS wire. CNA wire can be used instead of TMA wire because of its better range of action, high spring back, and less frictional resistance for space closure in sliding mechanics.

## Introduction

Orthodontic wires, which generate biomechanical forces, communicate through brackets for tooth movement and are key to the practice of orthodontics [1]. Friction is encountered whenever the archwire slides through the bracket. A portion of the applied force is thus lost, which ranges from 12% to 60%, resulting in decreased tooth movement. Consequently, more force needs to be applied to achieve the desired result. This causes excessive pain, loss of anchorage, and root resorption [2]. Thus, frictional forces generated between brackets and archwires should be minimized to allow optimal tooth movement [3].

Friction is defined as a force that delays or resists the relative motion of two objects in contact, and its direction is tangential to the common interface of the two surfaces [4]. There are two main types of friction: static friction, which prevents the motion, and kinetic (dynamic) friction, which occurs during the motion.

According to Nishio, et al., “Under normal conditions, the frictional force is proportional to the applied load, depending on the nature of the sliding surfaces, and independent of the contact area between the surfaces and the sliding speed (except at very low speeds). The frictional coefficient of a given material is the ratio between the tangential force and the normal or perpendicular load applied during the relative motion.” [5].

In recent years, Connecticut new arch (CNA) wire has been introduced into the market and has obtained popularity. However, there is limited information on CNA wires in previous orthodontic literature. Thus, the aim of this study was to evaluate static friction and surface roughness of 0.017 x 0.025 and 0.019 x 0.025-inch stainless steel (SS), titanium molybdenum alloy (TMA), and CNA wires in 0.02-inch slot SS and ceramic brackets.

## Materials and methods

The present study was carried out to compare the frictional resistance produced by pre-adjusted edgewise SS and ceramic brackets during sliding mechanics using SS, TMA and CNA wires. The SS and ceramic brackets used for the study were standard twin premolar brackets having four tie wings for engagement of elastomeric ligatures. Ethical clearance for the study was obtained from the institutional ethical committee. The study was conducted in the Department of Orthodontics, Government Dental College, Bengaluru, India. The tests were carried out in the Department of Nanotechnology and Research, Indian Institute of Sciences, Bengaluru, India. The duration of this study was three months, from August 2, 2018 to November 2, 2018.

One-hundred eighty premolar brackets each of SS (ORMCO Corp., Orange, CA) and ceramic (3M Unitek, Maplewood, MN) having a 0.022-inch slot and 180 each of SS, TMA and CNA wires of 0.017 x 0.025 inches and 0.019 x 0.025 inches were tested. The SS and ceramic brackets were bonded to the SS bar with cyanoacrylate adhesive with the help of a jig. Wire segments of 4 cm were ligated to the bracket with the help of modules. Each bracket-wire assembly was vertically mounted and clamped to the jaws of the universal testing machine (UTM) with 10 Newton load cell and friction was measured and readings were recorded. The surface roughness of brackets and wires was assessed at the SEM laboratory in the Mechanical Department in BMS College of Technology, Bengaluru, India. Surface roughness was assessed using an SEM at a magnification of 200 X before and after testing. Table 1 displays various brackets and wire combinations.

**Table 1:** Different bracket-wire combinations CNA, Connecticut new arch; TMA, titanium molybdenum

Groups	Bracket and Wire Combination
Group 1A	Stainless steel bracket and 0.017 x 0.025-inch stainless steel wire
Group 1B	Stainless steel bracket and 0.017 x 0.025-inch TMA wire
Group 1C	Stainless steel bracket and 0.017 x 0.025-inch CNA wire
Group 2A	Stainless steel bracket and 0.019 x 0.025-inch stainless steel wire
Group 2B	Stainless steel bracket and 0.019 x 0.025-inch TMA wire
Group 2C	Stainless steel bracket and 0.019 x 0.025-inch CNA wire
Group 3A	Ceramic bracket and 0.017 x 0.025-inch stainless steel wire
Group 3B	Ceramic bracket and 0.017 x 0.025-inch TMA wire
Group 3C	Ceramic bracket and 0.017 x 0.025-inch CNA wire
Group 4A	Ceramic bracket and 0.019 x 0.025-inch stainless steel wire
Group 4B	Ceramic bracket and 0.019 x 0.025-inch TMA wire
Group 4C	Ceramic bracket and 0.019 x 0.025-inch CNA wire

Descriptive statistics with mean and standard deviation were computed. Analysis of variance (ANOVA) and post hoc Tukey's test were used. Statistical significance was considered at *p *< 0.05 (confidence interval of 95% was taken).

## Results

**Figure 1:**
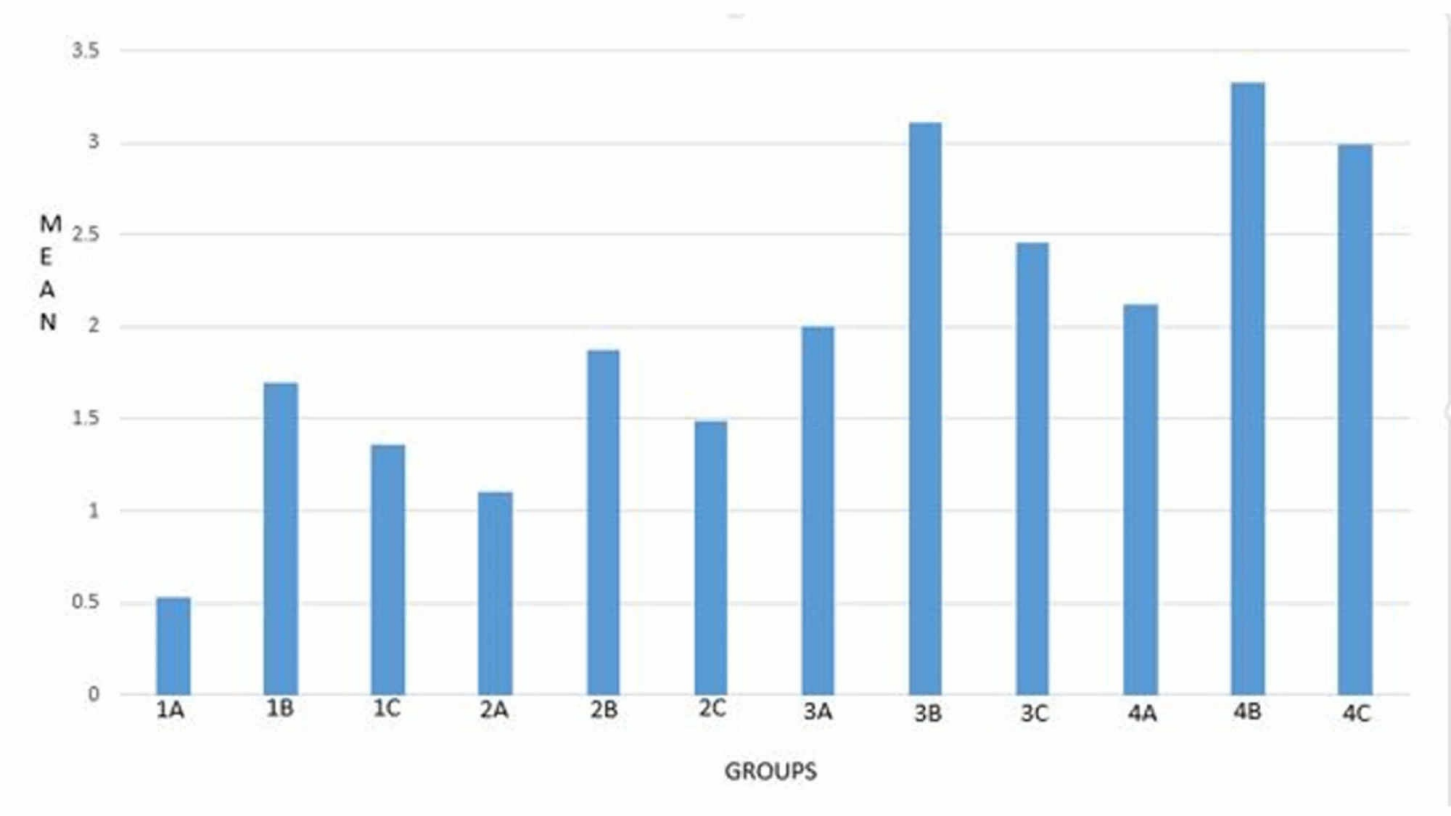
Mean static friction of bracket and wire combinations

The static friction obtained for various bracket-wire combinations is shown in Table 2, and a graphical representation is shown in Figure 1.

**Table 2:** Static friction (Newtons) of 12 groups CI, confidence interval; SD, standard deviation

Groups	Friction		
	Min-Max	Mean ± SD	95% CI
Group 1A	0.110-2.195	0.53 ± 0.40	0.38223-0.68677
Group 1B	1.800-3.710	1.70 ± 0.70	2.27906-2.65161
Group 1C	1.040-3.150	1.36 ± 0.61	1.73946-2.27321
Group 2A	0.520-1.600	1.10 ± 0.25	1.00839-1.19828
Group 2B	1.800-4.800	1.87 ± 1.01	3.01405-3.66328
Group 2C	0.710-2.350	1.49 ± 0.44	1.32987-1.65880
Group 3A	0.750-4.500	2.00 ± 0.71	1.50044-2.25623
Group 3B	1.060-6.620	3.11 ± 1.49	2.56075-3.67458
Group 3C	2.78825-3.19642	2.46 ± 0.49	2.310-4.140
Group 4A	0.490-2.050	2.12 ± 0.99	1.13698-1.59635
Group 4B	1.060-6.620	3.33 ± 0.86	2.56075-3.67458
Group 4C	0.870-3.730	2.99 ± 0.54	1.44802-1.97132

Tables 3, 4 and 5 show the results of various bracket-wire combinations.

**Table 3:** Comparison of friction of 0.017 x 0.025-inch stainless steel, titanium molybdenum alloy, and Connecticut new arch wires in stainless steel brackets **Strongly significant (P-value: P ≤ 0.01) CI, confidence interval.

	Mean difference	P-value	95% CI
Group 1A-1B	1.930833	0.000**	-2.61748 to -1.24419
Group 1A-1C	1.471833	0.000**	-2.15848 to -0.78519
Group 1B-1C	0.459000	1.000	-0.22764 to 1.14564

**Table 4:** Comparison of friction of 0.017 x 0.025-inch stainless steel, titanium molybdenum alloy, and Connecticut new arch wires in ceramic brackets **Strongly significant (P-value: *P* ≤ 0.01) CI, confidence interval

	Mean Difference	P-value	95% CI
Group 3A-3B	0.247000	1.000	-0.93364 to -0.43964
Group 3A-3C	1.114000	0.000**	-1.80064 to -0.42736
Group 3B-3C	0.867000^*^	0.002**	-1.55364 to -0.18036

**Table 5:** Comparison of friction of 0.019 x 0.025-inch stainless steel, titanium molybdenum alloy, and Connecticut new arch wires with stainless steel brackets **Strongly significant (P-value: *P *≤ 0.01) CI, confidence interval

	Mean Difference	P-value	95% CI
Group 2A-2B	2.235333	0.000**	-2.92198 to -1.54869
Group 2A-2C	0.391000	1.000	-1.07764 to 0.29564
Group 2B-2C	1.844333	0.000**	1.15769 to 2.53098

TMA wires showed the greatest frictional force compared to SS and CNA wires. The frictional force was greater in 0.019 x 0.025-inch wires compared to 0.017 x 0.025-inch wires. Among the various bracket-wire combinations, the highest frictional force was noted in the SS bracket and 0.019 x 0.025-inch TMA wire combination. A statistically significant difference was not seen between the SS bracket and 0.019 x 0.025-inch SS wire and 0.019 x 0.025-inch CNA wire combinations.

SEM examination indicated the ceramic bracket had a rougher surface compared to the SS bracket. The ceramic bracket showed a rough and irregular surface before testing, and deeper and wider craters were seen after testing. The SS bracket displayed a smooth surface with uneven patches before testing, and an irregular surface was seen after testing.

SEM examination indicated greater surface roughness of TMA wire compared to CNA and SS archwires. The SS wire had a smooth surface with regular striations before the testing, and deep striations were seen after the testing. The TMA wire had a rough surface with increased irregularities before testing, with increased and widened striations after testing. The CNA wire showed an irregular surface with vertical fissures before testing and deepening of the fissures after testing.

## Discussion

A combination of SS bracket and 0.017 x 0.025-inch archwire showed that friction was the least in the SS (0.53 ± 0.40) wire followed by CNA (1.36 ± 0.61) and TMA (1.70 ± 0.70) wire combinations. These findings were in concordance with those of Kapila et al. [6]. A combination of ceramic bracket and 0.017 x 0.025-inch wire showed that the friction was least with SS wire (2.00 ± 0.71) combination followed by CNA wire (2.46±0.49) and highest for TMA wire (3.11 ± 1.49) combination [5,7]. When comparing the above two combinations, the friction was lower in all the SS bracket-and-wire combinations than the ceramic bracket and wire combinations. The findings of this study are in concordance with the findings of Cash et al. [8].

When 0.019 x 0.025-inch wires were tested for friction in SS and ceramic brackets, it was found that friction was least with 0.019 x 0.025-inch SS wire and SS bracket (1.10 ± 0.25) combination and highest for the combination of TMA wire (3.33 ± 0.86) with ceramic bracket.

In the present study, we noted frictional forces increase when the size of the archwire increases; these findings are in accordance with the study by Kapila et al., Drescher et al., and Andreasen et al. [6,9-10]. Ceramic brackets showed the highest level of frictional resistance in all wire combinations. The significantly lower frictional resistance provided by the SS bracket is most likely a result of lower surface roughness [3,5,11]. According to Krishnan et al., TMA wires showed the highest frictional values in comparison with SS and CNA wires [12]. Thus, the net force required for translatory movement will be lower for SS and higher for TMA wires. Kusy and Whitley showed that TMA had a smoother surface than β-titanium wires, and the frictional resistance was similar to SS [13]. CNA wires had lesser surface roughness compared to TMA wires and slightly higher than SS wires. Thus, CNA wires had less friction compared to TMA wires and slightly higher friction than the SS wires.

In this present study, 0.019 x 0.025-inch CNA wires in SS and ceramic brackets showed almost similar frictional behavior to 0.019 x 0.025-inch SS wire so that CNA wire can be used instead of SS wire for retraction. However, since its stiffness is less than SS, torque expression may be affected [1].

**Figure 2:**
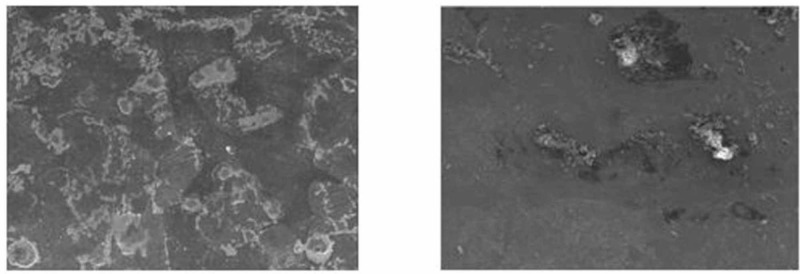
Photomicrography of SS bracket before (A) and after (B) friction testing SS, stainless steel

**Figure 3:**
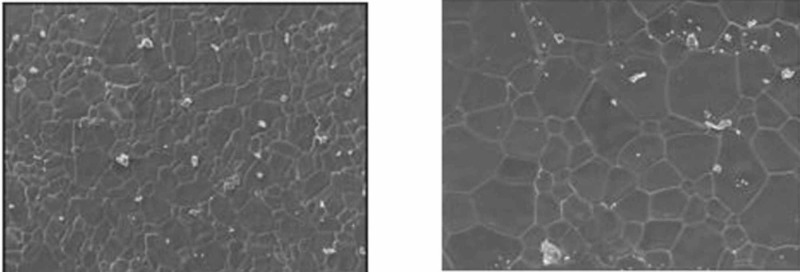
Photomicrography of ceramic bracket before (A) and after (B) friction testing

**Figure 4:**
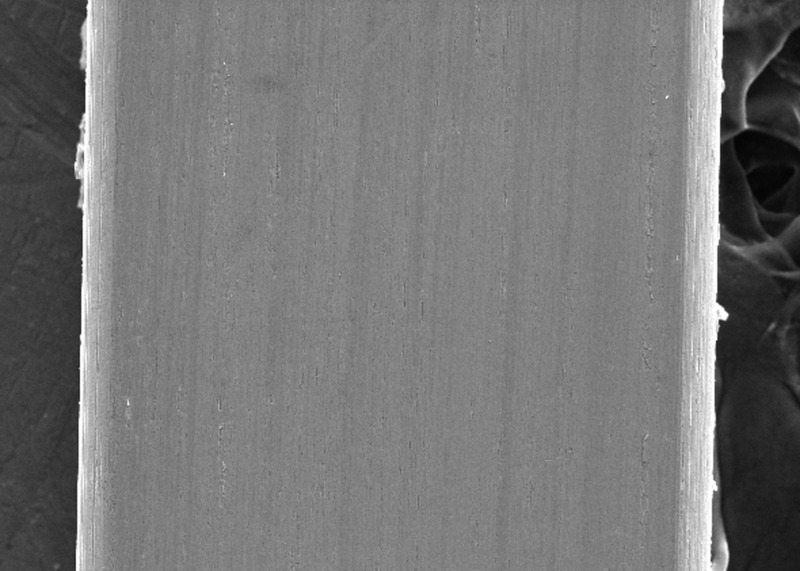
Photomicrography of SS wire had a smooth surface with regular striations before testing SS, stainless steel

**Figure 5:**
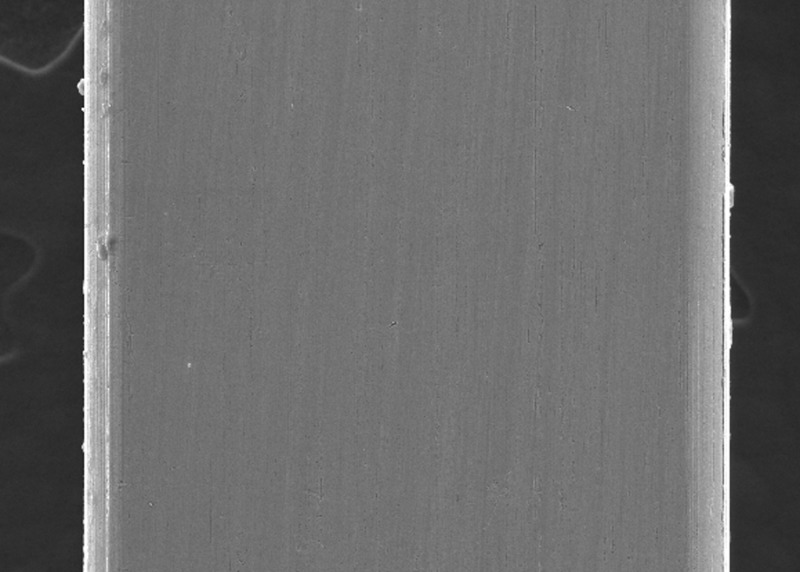
Photomicrography of SS wires showed deep striations after testing SS, stainless steel

**Figure 6:**
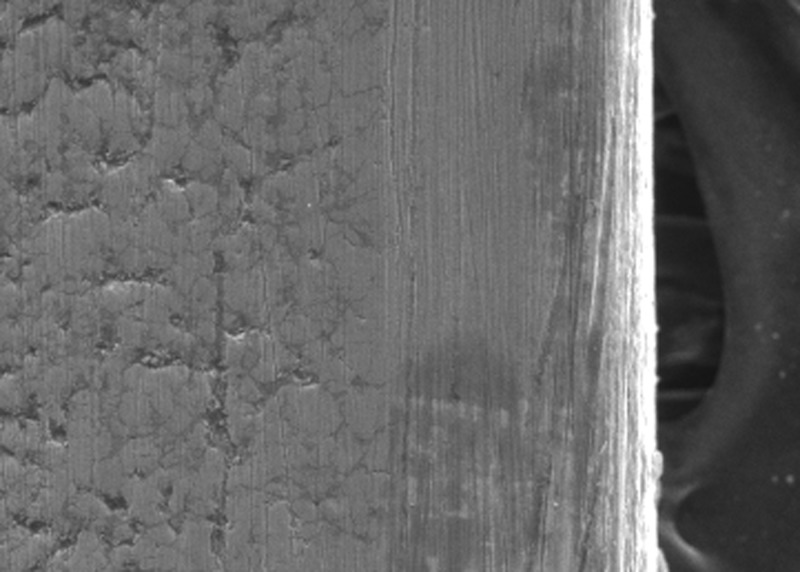
Photomicrography of TMA wires before testing TMA, titanium molybdenum archwire

**Figure 7:**
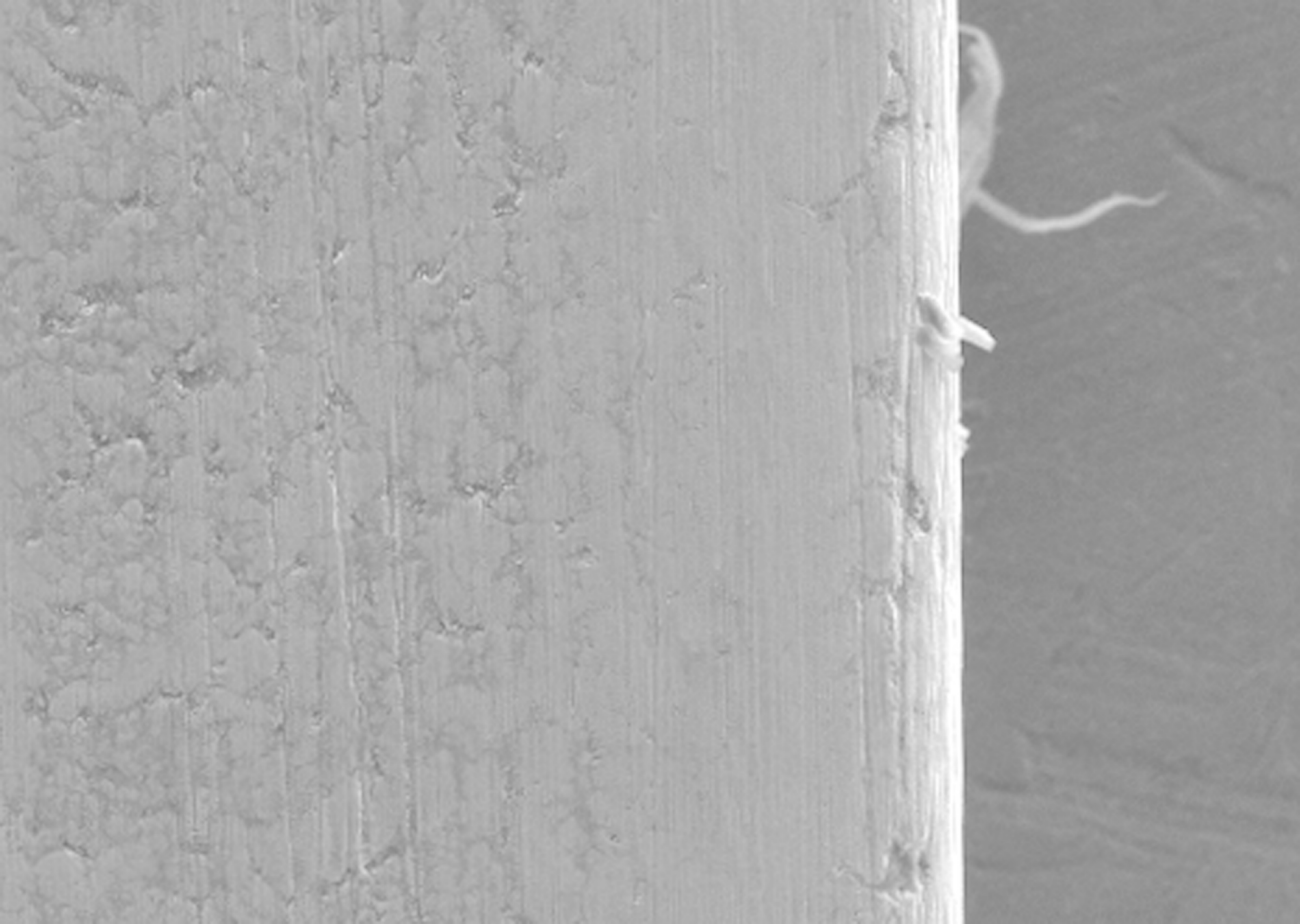
Photomicrography of TMA wire showed increased and widened striations after testing TMA, titanium molybdenum

**Figure 8:**
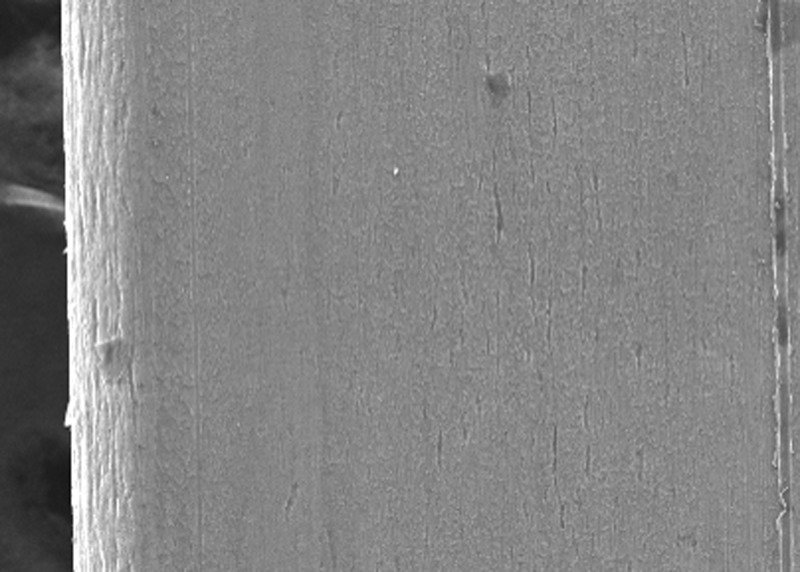
Photomicrography of CNA wire showed irregular surface with vertical fissures before testing CNA, Connecticut new arch

**Figure 9:**
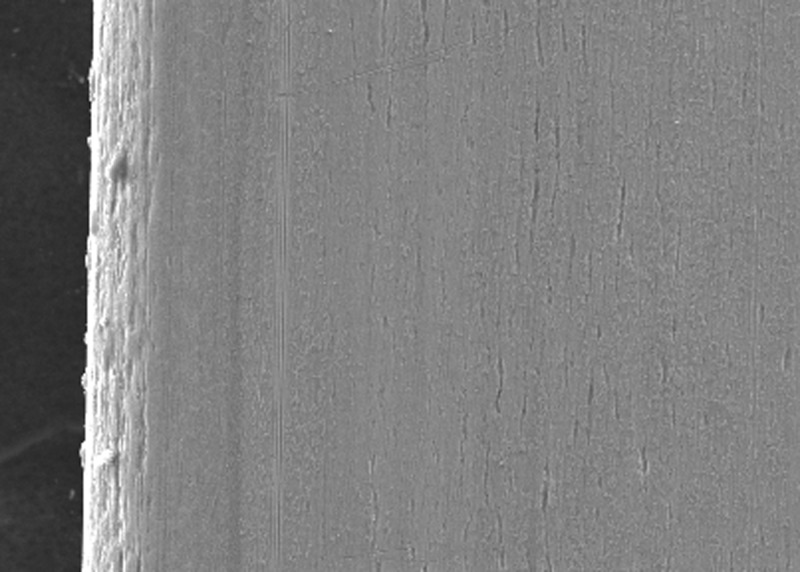
photomicrography of CNA wire showed deepening of the fissures after testing CNA, Connecticut new arch

In the present study, the surface roughness of brackets and wires was evaluated. The photomicrography of SS and ceramic brackets before(A) and after(B) friction testing is shown in Figures 2 and 3, respectively. The SS bracket had a smooth and regular surface, whereas the ceramic bracket showed a rough uneven surface. After friction testing, craters were seen on the surface of the SS bracket, and in the ceramic bracket, roughness increased with wide craters. Among the wires, the TMA wires showed a rougher surface than CNA and SS wires. The SS wire had a smooth surface (Figure 4) with regular striations before testing and deep striations (Figure 5) after testing. The TMA wire had a rough surface with increased irregularities (Figure 6) before testing and increased and widened striations (Figure 7) after testing. The CNA wire showed an irregular surface with vertical fissures (Figure 8) before testing, and the deepening of the fissures (Figure 9) was seen after testing. These findings align with the surface profilometry study done by Juvvadi et al., who observed that SS was the smoothest wire compared to CNA and TMA wires [1].

Krishnan et al. conducted an SEM evaluation of three wires (SS, TMA and Timolium) and showed that SS wires exhibited vertically oriented cracks, which can act as stress raisers, making the alloy more brittle. TMA showed a large number of uniformly distributed pores, exhibiting a very rough surface [12]. In the present study, similar surface characteristics were observed in SS and TMA wires.

Our study was limited in that only three types of archwires were compared at 10 N of load application by the UTM. Sometimes, SEM may show minute errors due to magnetic and electric vibrations interference. Future studies to test the friction between various esthetic archwires and different types of ceramic brackets using advance UTM are warranted.

## Conclusions

We compared the frictional force between three different archwire types and preadjusted edge-wise brackets. The surface roughness of the wires and brackets was also evaluated via SEM. The highest frictional force was noted in the SS bracket and 0.019 x 0.025-inch TMA wire combination due to its greater surface roughness. CNA wires had less frictional resistance than TMA wire and a similar frictional resistance as the SS wire. The SS wire had a smoother surface than CNA and TMA wires, resulting in lesser frictional resistance. The ceramic bracket showed more friction than the SS bracket as it had a rougher surface. A thorough knowledge about the friction between various archwire and bracket combinations enables the orthodontist to best select the appliance best suited to the patient’s needs, thus minimizing friction to deliver the best possible results. 
